# Screening Compounds with a Novel High-Throughput ABCB1-Mediated Efflux Assay Identifies Drugs with Known Therapeutic Targets at Risk for Multidrug Resistance Interference

**DOI:** 10.1371/journal.pone.0060334

**Published:** 2013-04-10

**Authors:** Megan R. Ansbro, Suneet Shukla, Suresh V. Ambudkar, Stuart H. Yuspa, Luowei Li

**Affiliations:** 1 Laboratory of Cancer Biology and Genetics, Center for Cancer Research, National Cancer Institute, Bethesda, Maryland, United States of America; 2 Laboratory of Cell Biology, Center for Cancer Research, National Cancer Institute, Bethesda, Maryland, United States of America; University of Pittsburgh, United States of America

## Abstract

ABCB1, also known as P-glycoprotein (P-gp) or multidrug resistance protein 1 (MDR1), is a membrane-associated multidrug transporter of the ATP-binding cassette (ABC) transporter family. It is one of the most widely studied transporters that enable cancer cells to develop drug resistance. Reliable high-throughput assays that can identify compounds that interact with ABCB1 are crucial for developing new therapeutic drugs. A high-throughput assay for measuring ABCB1-mediated calcein AM efflux was developed using a fluorescent and phase-contrast live cell imaging system. This assay demonstrated the time- and dose-dependent accumulation of fluorescent calcein in ABCB1-overexpressing KB-V1 cells. Validation of the assay was performed with known ABCB1 inhibitors, XR9576, verapamil, and cyclosporin A, all of which displayed dose-dependent inhibition of ABCB1-mediated calcein AM efflux in this assay. Phase-contrast and fluorescent images taken by the imaging system provided additional opportunities for evaluating compounds that are cytotoxic or produce false positive signals. Compounds with known therapeutic targets and a kinase inhibitor library were screened. The assay identified multiple agents as inhibitors of ABCB1-mediated efflux and is highly reproducible. Among compounds identified as ABCB1 inhibitors, BEZ235, BI 2536, IKK 16, and ispinesib were further evaluated. The four compounds inhibited calcein AM efflux in a dose-dependent manner and were also active in the flow cytometry-based calcein AM efflux assay. BEZ235, BI 2536, and IKK 16 also successfully inhibited the labeling of ABCB1 with radiolabeled photoaffinity substrate [^125^I]iodoarylazidoprazosin. Inhibition of ABCB1 with XR9576 and cyclosporin A enhanced the cytotoxicity of BI 2536 to ABCB1-overexpressing cancer cells, HCT-15-Pgp, and decreased the IC_50_ value of BI 2536 by several orders of magnitude. This efficient, reliable, and simple high-throughput assay has identified ABCB1 substrates/inhibitors that may influence drug potency or drug-drug interactions and predict multidrug resistance in clinical treatment.

## Introduction

ABCB1, also known as P-glycoprotein (P-gp) or multidrug resistance protein 1 (MDR1), is a membrane-associated multidrug transporter of the ATP-binding cassette (ABC) transporter family. ABCB1 is largely recognized for its role in enabling cancer cells to evade response to treatment via the efflux of chemotherapeutic agents. This multidrug resistance impedes the clinical cure of cancer by chemotherapy [1]. ABCB1 is also expressed in many normal cells and tissues, including the kidneys, liver, brain, intestine, and placenta, serving a key role in drug-drug interactions (DDI) [2] and the absorption, distribution, and excretion of a vast array of xenobiotics [3], [4]. For example, ABCB1 expressed in the intestine exports its substrates from intestinal epithelial cells to the luminal side of the intestine. The presence of an inhibitor for ABCB1 alters the bioavailability of a drug in the intestine and has an impact on the clinical safety of the selected drug [5]. To enhance current knowledge on the functional roles of ABCB1, to discover new compounds for cancer treatment, and to evaluate the interaction between ABCB1 and newly developed therapeutic agents, it is imperative to develop reliable assays that can efficiently and effectively characterize drug candidates.

Current *in vitro* methods used to elucidate the pharmacokinetics and dynamics of drug interactions with ABC transport proteins are carried out using either cell- or membrane-based assays. The cell-based assays employ cancer cell lines that have developed drug resistance [6] or cell lines that overexpress ABC transport proteins by drug selection or by means of plasmid transfection or viral vector transduction [7], [8]. Commonly used cell-based assays include either the direct measurement of drug transport across an epithelial cell (Caco-2 and MDCK) monolayer [9], [10] or an indirect measurement of transporter-mediated efflux of fluorescent substrates [10], [11]. Direct drug transport is also evaluated using inside-out plasma membrane vesicles isolated from cell lines overexpressing ABC transporters by measurement of drug transport into the lumen of these vesicles [12]. Another commonly used membrane-based assay tests if the drug interferes with ABCB1-ATPase activity [13], [14], [15]. In this assay, the ATPase activity of the ABC transporters is evaluated by either measuring the production of inorganic phosphate after ATP hydrolysis or by measuring remaining ATP with an ATP-dependent luciferase assay. The potential candidates for ABCB1 inhibition can also be determined based on their ability to interfere with the drug resistance of ABCB1-expressing cancer cell lines or compete for direct binding to the transporters [16], [17]. Though these assays have been used to evaluate ABCB1 substrates/inhibitors, such methods are not easily adaptable to high-throughput formats that would enable screening of large drug libraries.

ABC transporter activities can be measured in transporter-mediated fluorescent substrate efflux assays using either flow cytometry or fluorescent plate readers. Calcein AM, a cell-permeable, non-fluorescent compound, is a known ABCB1 substrate that has been used in flow cytometry assays for evaluating ABCB1 inhibitors or competitive substrates by measuring calcein AM efflux [18], [19], [20]. Hydrophobic calcein AM is rapidly diffused through plasma membranes and hydrolyzed by intracellular esterases to yield the highly fluorescent green anion, calcein, which is well retained in the cytoplasm of live cells. In ABCB1-overexpressing cells, the hydrophobic calcein AM is pumped out from the cell membranes by ABCB1, but retained in the cells in the presence of an ABCB1 inhibitor and then hydrolyzed to yield fluorescent calcein. The change in cellular fluorescence caused by the ABCB1 inhibitor is measured by flow cytometry [18]. A multiplex automated flow cytometry high-throughput assay has the sensitivity of the flow cytometry assay [21] and the ability to screen large libraries of compounds, but this custom-made system is not widely available. Fluorescent plate reader-based high-throughput efflux assays have also been used to screen ABC transporter inhibitors [22], [23], [24]. However, fluorescent plate readers are less sensitive than microscope-based cell imaging in cellular assays, since the plate reader is designed for homogenous assays [25]. High-throughput microscopy-based imaging systems are available and better equipped for cellular assays.

In this study, we describe the development and validation of a cell- and fluorescent imaging-based high-throughput assay to screen potential ABCB1 inhibitors and report the identification of multiple drug candidates that have not been previously known to interact with ABCB1. This assay was developed based on the same properties as the flow cytometry-based efflux assays that measure ABCB1-mediated efflux of calcein AM but has the advantage of being *in situ* cell-based, where cytotoxic effects can be directly monitored. It is easy to perform and requires no washing procedures. Our results demonstrate that this high-throughput assay is suitable for screening large numbers of natural and synthetic drug libraries to find potential ABCB1 inhibitors that could be used to advance disease treatment as well as enhance current biological and pharmacological knowledge on ABC transport proteins. The same approach may be applied to screen inhibitors of other ABC transporters with the use of transporter-expressing cell lines.

## Materials and Methods

### Chemicals

XR9576 (tariquidar) and fumitremorgin C (FTC) were gifts from Dr. Susan Bates (National Cancer Institute (NCI), Bethesda, MD). Bryostatin-1 was a gift from Dr. Peter Blumberg (NCI, Bethesda, MD). Calcein AM and MitoTracker® Green FM were purchased from Invitrogen (Carlsbad, CA). Cyclosporin A was purchased from LC Laboratories (Woburn, MA). A kinase inhibitor library consisting of 193 compounds and ispinesib (SB-715992) were purchased from Selleckchem Chemicals (Houston, TX). Verapamil, vinblastine, and dimethyl sulfoxide (DMSO) were purchased from Sigma-Aldrich (St. Louis, MO). MK-571 was purchased from Enzo Life Sciences, Inc. (Farmingdale, NY). IKK 16 was purchased from Tocris Bioscience (Minneapolis, MN). [^125^I]iodoarylazidoprazosin (IAAP, 2,200 Ci/mmol) was obtained from PerkinElmer Life and Analytical Sciences (Waltham, MA).

### Cell culture

ABCB1-overexpressing KB-V1 cells, previously selected and maintained with vinblastine (1 µg/ml), and the parental line, KB-3-1 cells, were cultured in DMEM supplemented with 10% FBS [26], [27], [28]. Vinblastine was removed from the KB-V1 cell culture medium 2 to 3 days before each experiment. For the efflux assay, the cells were plated at either 20,000 cells per well in 96-well (100 µl/well) or 2,500 cells per well in 384-well (40 µl/well) flat, clear bottom, white or black-walled polystyrene tissue culture plates (uncoated) (Corning Life Sciences, Tewksbury, MA) and incubated at 37°C. After the cell confluence reached 30–80% (1 to 3 days in culture), the cell and fluorescent imaging-based efflux assays were performed. The ABCB1 overexpressing HCT-15-Pgp cells, used for the cytotoxicity assays, were also cultured in DMEM supplemented with 10% FBS [29]. All cell lines were gifts from Dr. Michael M. Gottesman (NCI, Bethesda, MD).

### Immunoblotting

For detection of ABCB1 protein, KB-3-1 and KB-V1 cells were lysed in a buffer containing 25 mM Tris, pH 7.5, 150 mM NaCl, 250 mM sucrose, 1 mM EDTA, 1 mM EGTA, 100 μM PMSF, and then the lysates were sonicated. The particulate fractions were isolated by ultracentrifugation at 100,000×*g* for one hour then subjected to immunoblotting using an anti-ABCB1 antibody (C219, Fujirebio Diagnostic Inc., Malvern, PA). HRP-conjugated anti-mouse IgG was used as a secondary antibody (Cell Signaling, Danvers, MA). The immunoblots were visualized by Enhanced Chemiluminescence (Pierce, Rockford, IL), and the chemiluminescence signal was captured by an imaging system (ChemiDoc-It® Imager, UVP, LLC., Upland, CA).

### Cell and fluorescent imaging-based efflux assay

The cell- and fluorescent imaging-based ABCB1-mediated efflux assays were carried out using ABCB1-overexpressing KB-V1 cells and calcein AM. Calcein AM was diluted in the culture media and then added directly to the cells grown in 96- or 384-well plates at the indicated final concentrations. Additionally, MitoTracker® Green FM was used as a fluorescent substrate in the ABCB1-mediated efflux assay. MitoTracker® Green FM is a cell permeable, mitochondrial-selective probe that passively diffuses through cells and binds to mitochondrial proteins, enabling green-fluorescence detection via flow cytometry or fluorescent microscopy [30], [31]. To test their inhibitory activities on ABCB1-mediated efflux, XR9576, cyclosporin A, verapamil, and other compounds were diluted in culture medium and directly added to the cells before the addition of calcein AM or other fluorescent substrates. There was no incubation time after addition of the ABCB1 inhibitors. The only time delay between the addition of the inhibitors/drugs and the addition of calcein AM was the sample handling time. The total volume of culture medium per well for a 96-well plate was 200 µl, which included 100 µl of initial culture medium, 50 µl of the test compound (4x working stock solution), and 50 µl of calcein AM (4x working stock solution); the total volume per well for a 384-well plate was 60 µl, which included 20 µl of initial culture medium, 10 µl of the test compound (6× working stock solution), and 30 µl of calcein AM (2× working stock solution). All wells contained 0.1% DMSO; except when the drugs were serial-diluted, in which the wells with the highest drug concentration contained 0.1% DMSO and the rest of wells contained diluted DMSO from the same working stock solution. The cell culture vessels were placed in a fluorescent and phase-contrast live cell imaging system, the IncuCyte^TM^FLR (Essen BioScience, Ann Arbor, MI), in a 37°C incubator supplemented with 5% CO_2_. Both fluorescent and phase-contrast images (750×950 µm) were taken by the IncuCyte^TM^FLR at the indicated time intervals. The approximate number of cells scanned was 200–600 cells per image. Auto-fluorescence of the compounds was determined by treating the cells with the drug candidates in the absence of calcein AM and fluorescence was measured using the IncuCyte^TM^FLR and a fluorescent plate reader (Infinite 200 PRO, Tecan System, Inc., San Jose, CA). Compounds that auto-fluoresced were excluded from further analysis.

The IncuCyte^TM^FLR was programmed to take 4 images per well of a 96-well plate or a single image per well of a 384-well plate. It takes 27 minutes to scan an entire 96-well plate at 4 images per well and 27 minutes to scan an entire 384-well plate at one image per well, respectively. Because intracellular accumulation of calcein is time sensitive, only 3 to 6 columns of a 96-well plate were used for a single experiment. Three positive control (XR9576) wells and three background (calcein AM only) wells were included in each 96-well plate or in each column in the 384-well plates. All experiments were repeated at least twice unless otherwise indicated.

### Flow cytometry-based efflux assay

An ABCB1-mediated calcein AM efflux assay was also performed with KB-V1 cells and evaluated by flow cytometry as described previously [32]. KB-V1 cells were detached by brief trypsin treatment, followed by addition of medium containing 10% serum to neutralize the trypsin. The detached cells were collected by centrifugation, aliquoted at 500,000 cells/tube, and incubated with the test compounds at the indicated concentrations and calcein AM (1 µM) or MitoTracker® Green FM for 10 minutes in a 37°C water bath. After incubation, the cells were collected by centrifugation at 800x*g* for 5 minutes and resuspended in PBS/0.1% BSA. The fluorescent substrates retained in KB-V1 cells were detected by flow cytometry (BD FACSCalibur, BD Biosciences, Franklin Lakes, NJ). The data were analyzed with FlowJo 7.2.2 (Tree Star, Inc., Ashland, OR) and presented as histograms. The percentage of the maximum (% of Max) is the number of cells in each bin divided by the number of cells in the bin that contains the largest number of cells (256 bins total). Total number of cells counted per sample was 10,000.

### Photolabeling of ABCB1 with [^125^I]IAAP

The interaction of selected compounds with ABCB1 was assessed by an *in vitro* photolabeling assay as previously described [32]. Crude membranes from Hifive cells overexpressing human ABCB1 were incubated with the selected compounds for 5 minutes, after which 3 to 5 nmol/L [^125^I]IAAP in 50 mM Tris-HCl (pH 7.5) was added. After exposure to UV light (366 nm) for 10 minutes at room temperature to covalently crosslink [^125^I]IAAP with ABCB1, the samples were separated by electrophoresis, and gels were dried and exposed on Bio-Max MR film (Eastman Kodak Company, Rochester, NY).

### Cell viability assay

HCT-15-Pgp cells were plated and cultured in 96-well plates for 1 or 2 days, until they reached 50–80% confluency. The cells were treated with the indicated concentrations of BI 2536, cyclosporin A, and XR9576 for 48 hours. Cell viability was examined using the Cell Counting Kit-8 (CCK-8, Dojindo Molecular Technologies, Inc., Rockville, MD); which functions similar to MTT assays by reduction of the tetrazolium salt, WST-8, to yield a yellow-colored and water-soluble formazan dye (measured at 450 nm).

### Data analysis

The IncuCyte^TM^FLR software provides the mean of the total fluorescence intensity (mean fluorescence intensity), obtained by averaging fluorescent intensities from all the pixels in the image(s) from each well. The automatically generated mean fluorescence intensity is not corrected for the background fluorescence, which can often lead to distorted outputs. Additional software, the Object Counting v2.0 Analysis software provided by the IncuCyte^TM^FLR, can assist further quantification and analysis of the fluorescent images. Using this software, a segmentation mask was generated to separate fluorescent (foreground) and non-fluorescent (background) objects so that the sum of the fluorescent intensity of the positive cells can be calculated without the interference from the background fluorescence. The Object Summed Intensity per mm^2^ from the metrix menu was chosen to indicate the background corrected fluorescent intensity of each well and designated as the object intensity.

The relative inhibition of each compound on calcein AM efflux was calculated by the following equation:
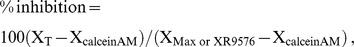
where X represents the mean fluorescence intensities or the object intensities, and T denotes the test compound. The final results were normalized to either the maximum values of the test compounds in the dose-response experiments or to the values from XR9576 (0.2 µM) treated cells. The IC_50_ values and Hill slopes of the dose-response curves were calculated using nonlinear regression analysis with variable slopes (4 parameters), GraphPad Prism 5 (GraphPad Software, Inc., La Jolla, CA). The Z-factor, a parameter that reflects both the assay signal dynamic range and the data variation [33], was calculated by the equation:



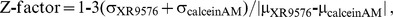
where σ and µ represent the standard deviations and means, respectively. Values from calcein AM-treated cells were set as background, and values from XR9765/calcein AM-treated cells were set as the positive control.

A *t*-test was performed to evaluate if the two groups of data were significantly different or not as indicated by the *p* values (GraphPad Prism). Among the three independent experiments, correlation coefficients between any two data sets were calculated in MS Excel and the three data sets were plotted in 3D scatter graphs using SigmaPlot (Systat Software, Inc., San Jose, CA).

## Results

### Assay set-up and optimization

**Figure 1:**
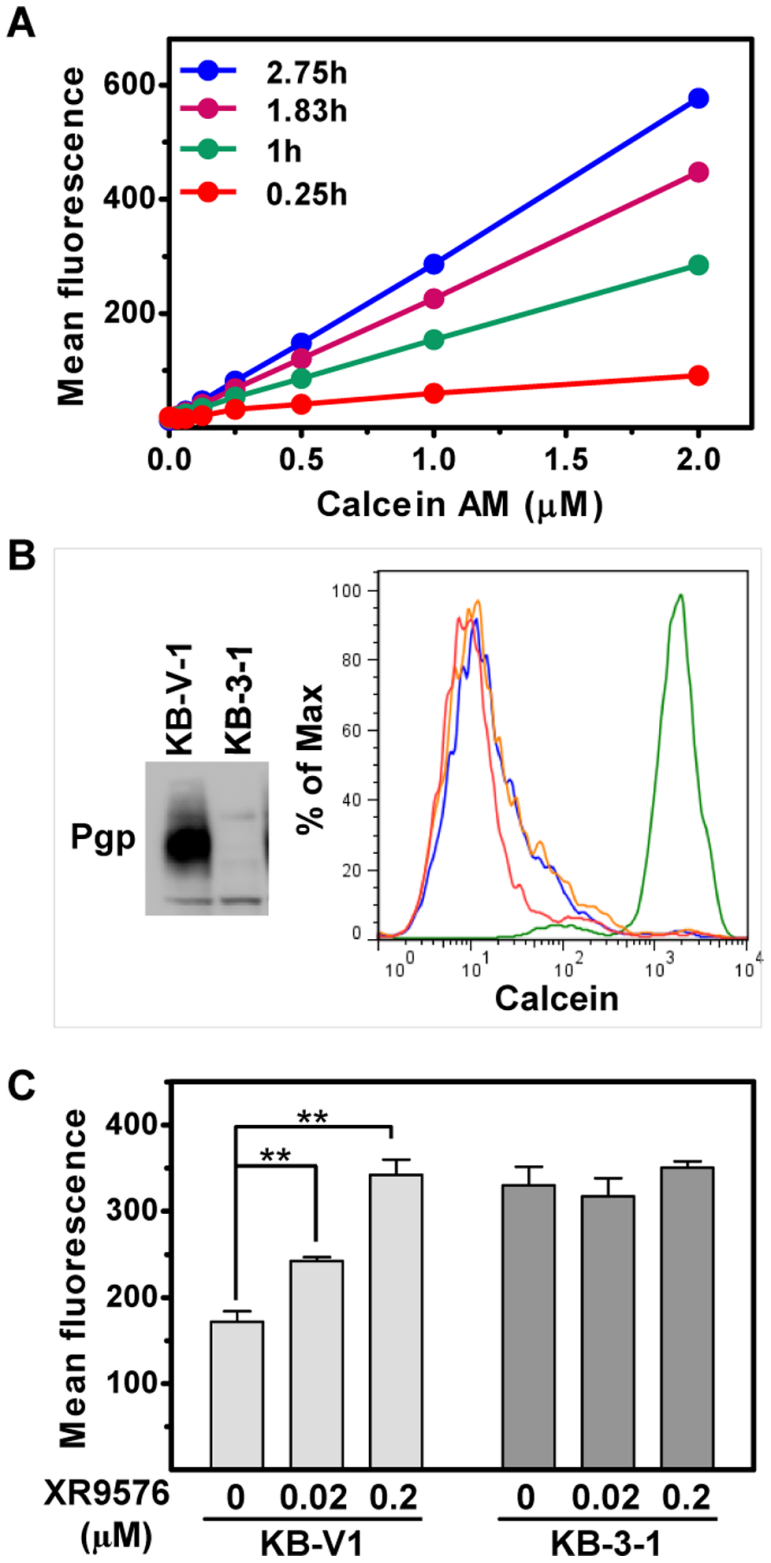
Cell imaging-based ABCB1-mediated calcein AM efflux by KB-V1 and KB-3-1 cells. **A.** Time-and dose-dependent fluorescent calcein accumulation in KB-V1 cells. Various concentrations of calcein AM were added to KB-V1 cells. The phase-contrast and fluorescent images were taken at the indicated times by the IncuCyte^TM^FLR. The mean fluorescence intensities were obtained from the IncuCyte^TM^FLR and plotted. Data presented are mean ± SD (n = 3). **B.** KB-V1 cells overexpress ABCB1. Particulate fractions of KB-3-1 and KB-V1 cells were subjected to western blotting with an anti-ABCB1 antibody. For the flow cytometry efflux assay, KB-V1 cells were treated with 5 µM of each inhibitor followed by 1 µM calcein AM. Red is for calcein AM; green is for XR9576; blue is for FTC; orange is for MK-571. **C.** XR9576 enhances calcein fluorescence in KB-V1 cells but not KB-3-1 cells. KB-V1 and KB-3-1 cells were treated with XR9576 before addition of calcein AM (1 µM). The phase and fluorescent images were taken 1 hour later by the IncuCyte^TM^FLR. The mean fluorescence intensities were obtained from the IncuCyte^TM^FLR. Data presented are mean ± SD (n = 3). ** indicates *p*<0.05.

To evaluate cellular accumulation of fluorescent calcein in KB-V1 cells (ABCB1-overexpressing) and KB-3-1 cells (parental cell line), the IncuCyte^TM^FLR imaging technology, capable of recording phase-contrast and fluorescent images from 96- and 384-well plates, was used. After KB-V1 cells grown in 96-well plates were incubated with increasing concentrations of calcein AM, the fluorescent and phase-contrast images were taken by the live cell imaging system at various time points. As shown in Figure 1A, the cellular fluorescence intensity, resulting from intracellular accumulation of fluorescent calcein, is positively correlated to the calcein AM concentrations in the culture medium. Accumulation of calcein in KB-V1 cells was also time-dependent (Figure 1A). To confirm that calcein AM efflux in KB-V1 cells is due to the overexpression of ABCB1, cell lysates from KB-3-1 and KB-V1 cells were subjected to immunoblotting with an anti-ABCB1 antibody. Figure 1B showed that only KB-V1 cells expressed detectable ABCB1 protein. The flow cytometry assay also indicated that the ABCB1 specific inhibitor, XR9576, blocked calcein AM efflux in KB-V1 cells, but neither ABCG2 specific inhibitor FTC nor ABCC1 specific inhibitor MK-571 interfered with ABCB1-mediated calcein AM efflux in KB-V1 cells (Figure 1B), suggesting that ABCC1 and ABCG2 are not involved in calcein AM efflux in KB-V1 cells. To further evaluate the cell imaging-based efflux assay, KB-V1 and KB-3-1 cells were compared. As shown in Figure 1C, KB-V1 cells retained less fluorescent calcein than KB-3-1 at 1 µM of calcein AM after one hour. The presence of XR9576 enhanced the total cellular fluorescent calcein accumulation in KB-V1 cells. In contrast, the fluorescent calcein accumulation in KB-3-1 cells was independent of XR9576 (Figure 1C). These results confirm the previous finding that ABCB1 is responsible for calcein AM efflux in KB-V1 cells [26], [27]. The efflux of MitoTracker® Green FM, another ABCB1 substrate [34], is also blocked by XR9576 in the flow cytometry assay, but the cellular fluorescence was much less intense than calcein (Figure S1A). The lower cellular fluorescence of MitoTracker® Green FM also reflected in the cell imaging-based efflux assay using the IncuCyte^TM^FLR (Figure S1B & C and see below). The effect of DMSO, a solvent for all of the compounds, on ABCB1-mediated efflux of calcein AM (1 µM) was evaluated, and our results indicate that DMSO is not auto-fluorescent, but is toxic to KB-V1 cells at 0.5% (v/v) and above (data not shown).

These results indicated that ABCB1 is the only ABC transporter that mediates calcein AM efflux in KB-V1 cells and that only intracellular-fluorescent substrates of ABCB1 are suitable for this cell imaging-based efflux assay. The data demonstrate that the IncuCyte^TM^FLR platform can be used to monitor calcein AM efflux mediated by ABCB1, based on analysis of cell- and fluorescent images of KB-V1 cells.

### Comparing raw data vs. background corrected data from the IncuCyte^TM^FLR

**Figure 2:**
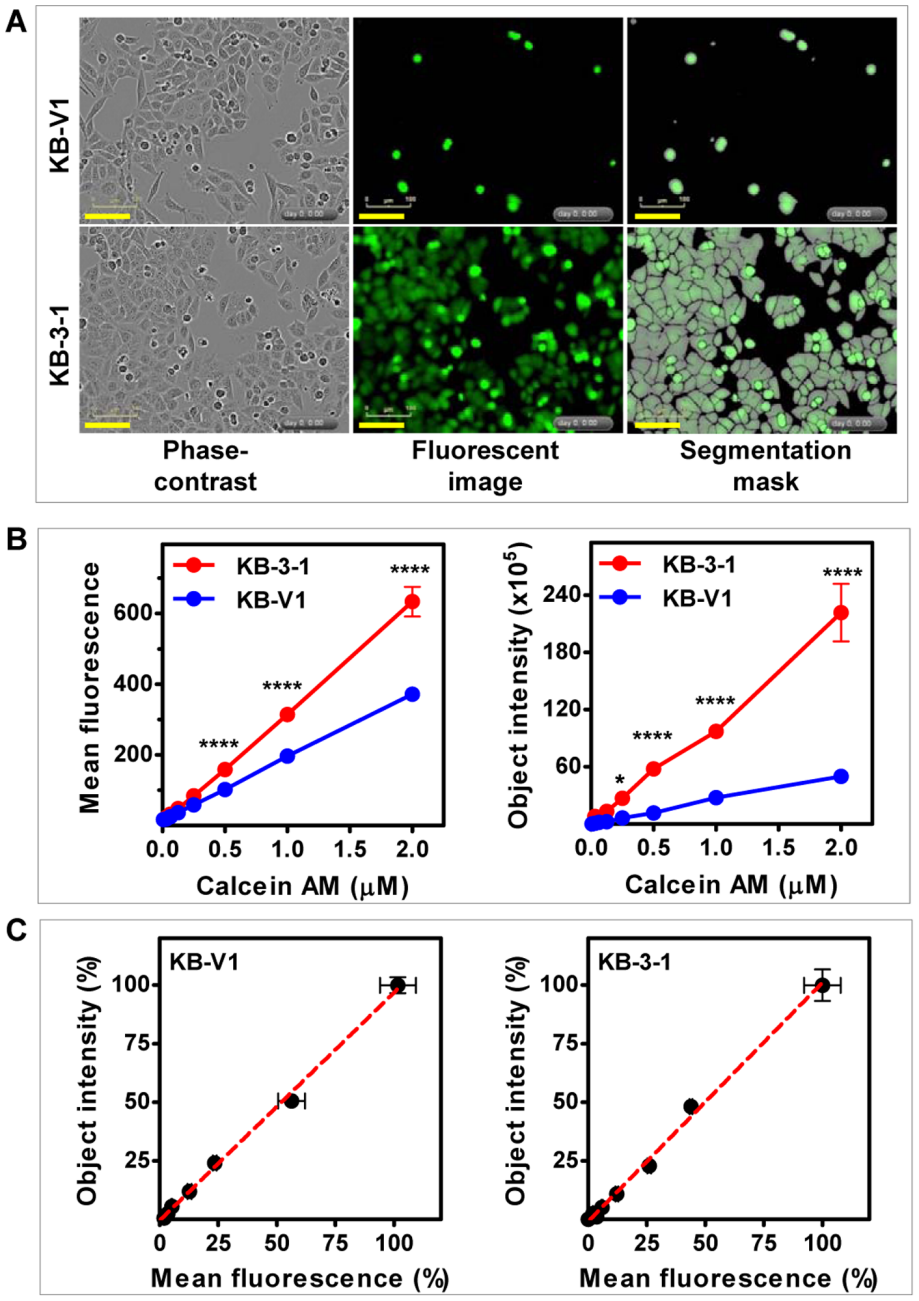
Analysis of fluorescence data from cell imaging-based efflux assay. **A.** Calcein fluorescence in KB-V1 and KB-3-1 cells. Calcein AM (1 µM) was added to KB-V1 and KB-3-1 cells. One hour later, phase-contrast and fluorescent images were taken by the IncuCyte^TM^FLR. Phase-contrast, fluorescent, and segmentation mask images are displayed (bar = 100 µm). **B.** Calcein AM dose-dependent fluorescence of KB-V1 and KB-3-1 cells. KB-3-1 and KB-V1 cells were loaded with various concentrations of calcein AM for 1 hour. The mean fluorescence intensities and the object intensities derived from the fluorescent images were exported from the IncuCyte^TM^FLR and graphed. * indicates the *p* value is less than 0.05; **** indicates the *p* value is less than 0.0001. **C.** Correlation between normalized florescence mean intensities and the object intensities. The data sets in Fig. 2B from KB-V1 and KB-3-1 cells were normalized within each data set and plotted. The red lines in the graph are the best fit lines for each data set analyzed for linear correlation. All data presented are mean ± SD (n = 3).

The phase-contrast and fluorescent images (Figure 2A) revealed that, at 1 µM calcein AM, only a fraction of KB-V1 cells were positive for fluorescence; in contrast, nearly all KB-3-1 cells were fluorescent at the same calcein AM concentration. At 1 µM calcein AM, the mean fluorescence intensities, available from the IncuCyte^TM^FLR, for KB-V1 and KB-3-1 cells were 159.3 and 370.4, respectively. The mean fluorescence intensity of KB-V1 cells was 52.4% of the KB-3-1 cells. Using the Object Counting v2.0 software, the fluorescent positive cells were masked as objects, as shown in Figure 2A (segmentation mask). The object intensity was calculated by subtracting the background fluorescence value (in the unmasked area) from the total fluorescence value of each image. The newly calculated object intensites for KB-V1 and KB-3-1 were 370.4 and 10,503.9, respectively. The object intensity of KB-V1 cells was only 3.52% of the KB-3-1 cells. The mean fluorescence intensities and the background object intensities from KB-V1 and KB-3-1 cells were plotted and displayed in Figure 2B. As shown in Figure 2B (left graph), the mean fluorescence intensities of KB-V1 and KB-3-1 cells are significantly different at 0.5, 1, and 2 µM calcein AM. In comparison, the right graph (Figure 2B) shows that the object intensities between KB-V1 and KB-3-1 cells are also significantly different at 0.25 µM calcein AM, a dosage at which differences in the mean fluorescence intensities between KB-V1 and KB-3-1 were indistinguishable. Results from the MitoTracker® Green FM efflux experiment showed that XR9576 inhibition on ABCB1-mediated efflux was detected when background fluorescence was subtracted (Figure S1C), but the results displayed no difference when the data from the mean fluorescence intensities were plotted (Figure S1B). These results demonstrate that background correction using the Object Counting v2.0 software is helpful when samples have low fluorescent signals. The correlations between the raw data set (the mean fluorescence intensities) and the background subtracted data set (the object intensities) from KB-V1 and KB-3-1 cells were evaluated. The two data sets were first normalized to the maximum value of each set and then plotted as the relative mean fluorescence intensity (%) (X-axis) vs. the relative object intensity (%) (Y-axis). As shown in Figure 2C, both sets of data from KB-V1 and KB-3-1 cells are significantly correlated to each other (*p*<0.0001 and R^2^ = 0.99), suggesting the raw data obtained from the mean fluorescence intensities without background subtraction can be used for the IncuCyte^TM^FLR-based ABCB1-mediated high-throughput efflux assay when calcein AM is used in the imaging-based assay.

### Evaluating ABCB1 inhibitors, XR9576, verapamil, and cyclosporin A, using the cell imaging-based efflux assay

**Figure 3:**
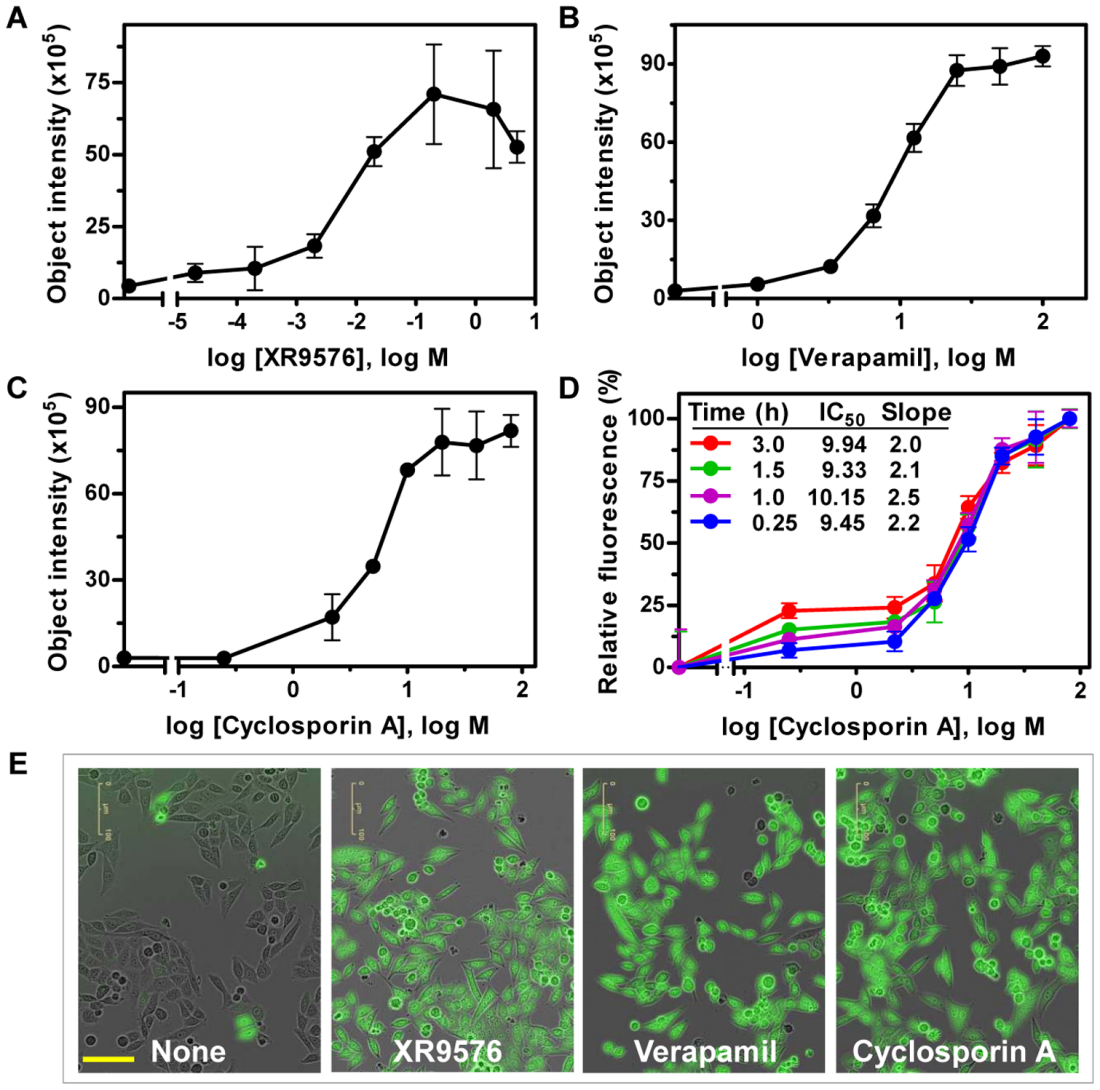
Evaluation of XR9576, verapamil, and cyclosporin A in the ABCB1-mediated efflux assay using the IncuCyte^TM^FLR. **A,** **B,** & **C.** KB-V1 cells were treated with the indicated compounds at various concentrations and then treated with 1 µM calcein AM. Images were taken 1 hour later. Object intensities were calculated and plotted. All data presented are mean ± SD (n = 3). **D.** KB-V1 cells treated with increasing cyclosporin A concentrations and 1 µM calcein AM were imaged at the indicated time points. The mean fluorescence intensities were normalized within the same time point to the highest concentration of cyclosporin A. The IC_50_ and Hill slope values were calculated for each time point as described in the Materials and Methods. All data presented are mean ± SD (n = 3). **E.** XR9576, verapamil, and cyclosporin A enhanced calcein fluorescence in KB-V1 cells. KB-V1 cells treated with the indicated compounds and 1 µM calcein AM were imaged 1 hour later. The phase-contrast and fluorescent images were merged and displayed (size bar = 100 µm).

XR9576, verapamil, and cyclosporin A are well-documented ABCB1 substrates/inhibitors [35], [36], [37], [38]. To test the inhibitory effect of these compounds on ABCB1-mediated efflux using the IncuCyte^TM^FLR, KB-V1 cells grown in 96-well plates were treated with increasing concentrations of each compound and then incubated with 1 µM calcein AM. Phase-contrast and fluorescent images (4 images per well) were acquired one hour after the initial addition of calcein AM. The fluorescent images were further analyzed using the Object Counting v2.0 software to remove the background fluorescence. As shown in Figure 3A, B, and C, XR9576, verapamil, and cyclosporin A displayed dose-dependent inhibition of ABCB1-mediated calcein AM efflux. The IC_50_ values for XR9576, verapamil, and cyclosporin A are 7.28 nM, 9.45 µM, and 5.57 µM, respectively. XR9576 was cytotoxic to cells above concentrations of 1 µM (not shown). The effect of cyclosporin A on ABCB1-mediated efflux was also evaluated at different time points after the addition of calcein AM. Figure 3D shows the normalized mean fluorescence intensities plotted at each time point. The dose-response curves of cyclosporin A at each time point displayed similar IC_50_ values and Hill slopes, suggesting that consistent results can be obtained even when the fluorescent images are taken at different time points, as long as the images from both positive and negative controls are taken at the same time. Merged phase-contrast and fluorescent images (Figure 3E) showed that in the absence of any inhibitors, few KB-V1 cells were positive for calcein fluorescence. Treatment with XR9576 (0.2 µM), verapamil (10 µM), and cyclosporin A (10 µM) increased the percentage of KB-V1 cells that were positive for intracellular fluorescent calcein. These results confirmed that the IncuCyte^TM^FLR fluorescent live cell imaging system is effective and efficient for high-throughput screening of ABCB1 inhibitors with a wide range of dosages at desired time points.

The fluorescent live cell imaging-based assay and the fluorescent plate reader-based efflux assays were directly compared using calcein AM and verapamil. The dose-response curves of verapamil inhibited-calcein AM efflux were similar between the two assays (Figure S2). The quality of the cell imaging-based efflux assay was also evaluated by calculating the Z-factor, which is reflective of both the assay signal dynamic range and the data variation [33], using calcein AM-treated cells as background and XR9765/calcein AM-treated cells as positive samples. The Z-factors, calculated from three positive control wells (XR9576 treated cells) and three negative control wells (calcein AM only treated cells), ranged between 0.5 and 0.84, in six independent experiments using 96-well plates, indicating that the IncuCyte^TM^FLR-based ABCB1-mediated efflux assay would be an excellent high-throughput assay when 96-well plates are used.

### Reproducibility of the cell imaging-based ABCB1-mediated efflux assay

**Figure 4:**
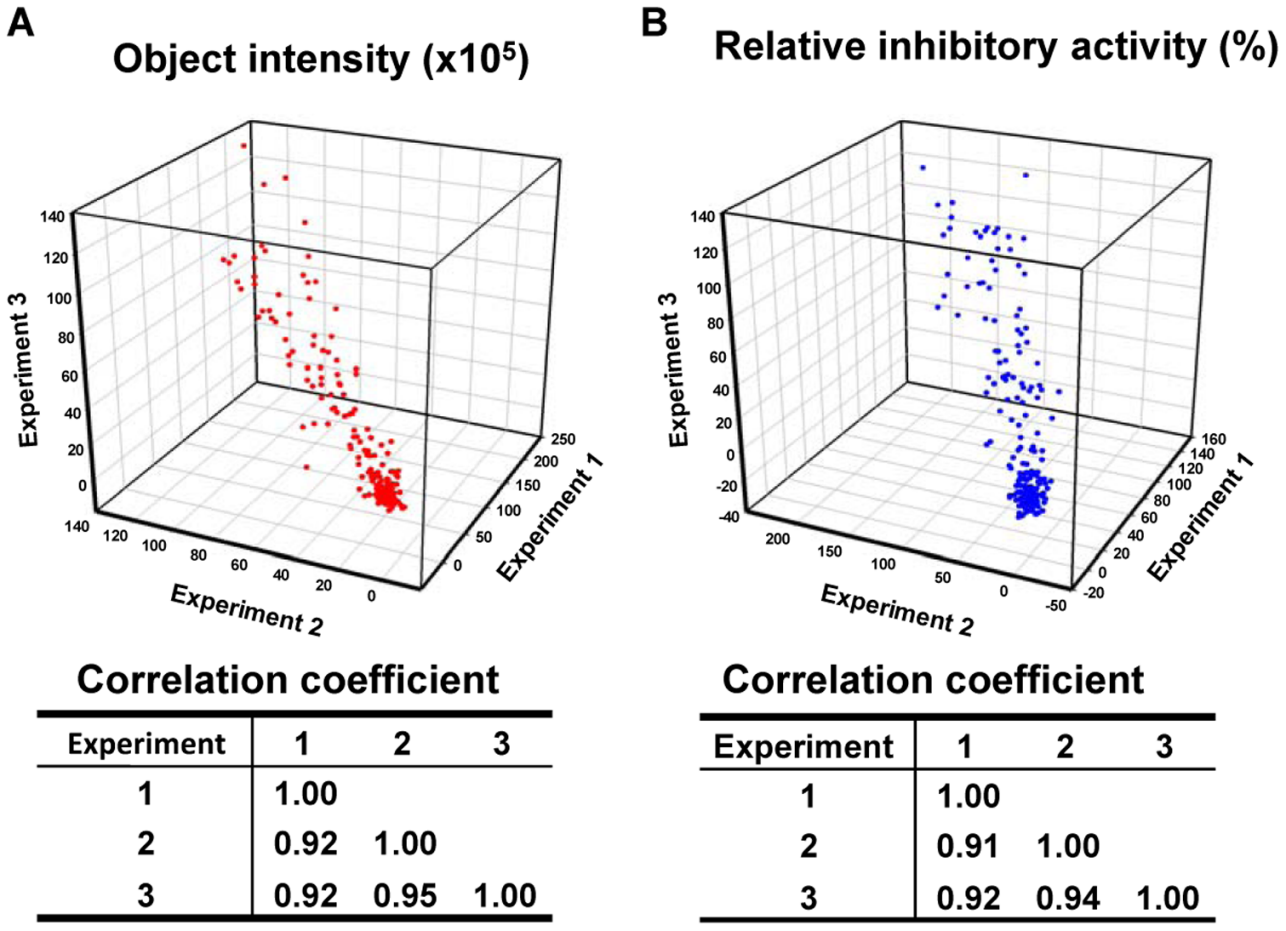
The fluorescent cell imaging-based ABCB1-mediated efflux assay is reproducible. Three independent experiments were performed to screen the inhibitor library in the cell imaging-based ABCB1-mediated calcein AM efflux assay in 384-well plates. The final concentration of each compound was 10 µM. The object intensities (A) and the relative inhibitory activities (B) of each compound were calculated. 3D scatter graphs were plotted using SigmaPlot. The correlation coefficients between any two data sets were calculated in MS Excel.

To test the reproducibility and large screening capacity of the cell- and fluorescent imaging-based high-throughput ABCB1-mediated efflux assay, KB-V1 cells were plated in 384-well plates and treated with the kinase inhibitor library of 193 compounds and calcein AM, then imaged using the IncuCyte^TM^FLR. Three independent experiments were performed. Three positive controls, cells treated with XR9576/calcein AM, and three negative controls, cells treated with calcein AM only, were included in each column of the 384-well plate. The relative object intensity of each well was calculated as described in the Materials and Methods section by normalizing the object intensities of the test compounds to XR9576 (0.2 µM, 100%) treated KB-V1 cells (three wells) in the same column. The background levels in each column were determined by the average object intensities of calcein AM-treated cells (three wells) in the same column. The object intensities and the relative ABCB1 inhibitory activities from three independent experiments were plotted as 3D scatter graphs as shown in Figure 4. The correlations between any two experiments were analyzed and also displayed in Figure 4. The results indicated that the three experiments are strongly correlated to each other. The Z-factors for the 384-well plates were also calculated between the positive and the negative controls in each column, as shown in Figure S3. The median value of Z-factors is 0.54. The Z-factors from the 384-well plate assays showed a wide distribution: 57% of the Z-factors are larger than 0.5, indicating an excellent assay; 31% of Z-factors are between 0 and 0.5, indicating a marginal assay. The remaining 12**%** of the Z-factors are less than 0. Compared to the 96-well plate assay, the 384-well plate assay is less robust, which is largely caused by the variation in cell density, since only a single image per well is recorded in a 384-well plate assay; in contrast, four images per well are recorded and averaged in a 96-well plate. These results indicate that the data produced by the IncuCyte^TM^FLR for the ABCB1-mediated efflux assay are highly reproducible in the 384-well plate format and suggest that it is a suitable high-throughput assay for libraries containing large numbers of compounds.

### Identification of BEZ235, BI 2536, and IKK 16 as ABCB1 inhibitors

**Figure 5:**
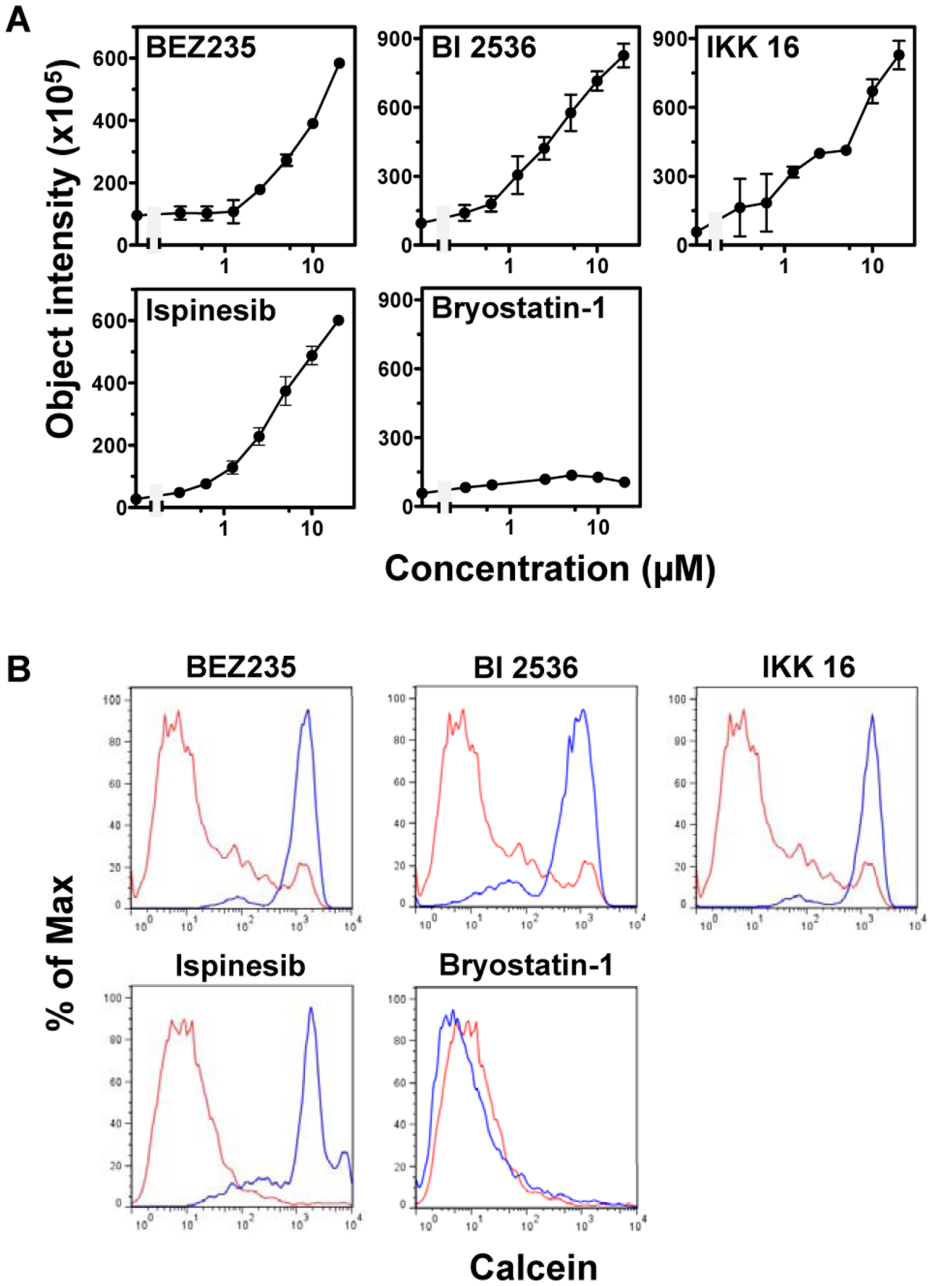
BEZ235, BI 2536, IKK 16, and ispinesib are inhibitors of ABCB1-mediated efflux. **A.** KB-V1 cells were treated with BEZ235, BI 2536, IKK 16, ispinesib, and bryostatin-1 and then calcein AM (1 µM). Doses of each compound were generated from 1∶2 dilutions of the highest concentration of 20 µM. The object intensities were plotted. Data presented are mean ± SD (n = 3). **B.** Flow cytometry-based efflux assays were performed to examine KB-V1 cells incubated with either calcein AM (1 µM, red line) or calcein AM plus BEZ235, BI 2536, IKK 16, ispinesib, or bryostatin-1 (5 µM, blue line).

The results from screening the inhibitor library of 193 total compounds, described in the previous section, were further analyzed. Positive hits were defined as compounds displaying at least 50% inhibition of calcein AM efflux in three repeats where XR9576 was used as positive control (100% inhibition). A total of 36 compounds were identified as inhibitors for ABCB1-mediated calcein AM efflux (Table 1). Thirteen of the inhibitors (marked * in Table 1) have previously been shown to interact with ABCB1 (referenced in Text S1) which further validates the usefulness of the IncuCyte^TM^FLR platform. However, the majority (63.9%) of newly identified ABCB1 inhibitors from this screen have never been previously reported to interact with ABCB1 (Table 1). BEZ235 and BI 2536 from the kinase inhibitor library and IKK 16 and ispinesib, identified from other screening assays, were further validated. Seven-point serial dilutions of each compound were tested in the cell- and imaging-based efflux assay in 96-well plates, and the dose-response curves for each compound are displayed in Figure 5A. The IC_50_ values for BEZ235, BI 2536, and ispinesib were 20.1, 3.92, and 5.04 µM, respectively; the IC_50_ value for IKK 16 cannot be calculated from the data. The flow cytometry-based ABCB1-mediated calcein AM efflux assays were performed to confirm that the four compounds are ABCB1 inhibitors (Figure 5B). Bryostatin-1, a compound that did not exhibit any inhibitory activity toward ABCB1-mediated efflux in the IncuCyte^TM^FLR-based efflux assay, was also further evaluated with the flow cytometry-based calcein AM efflux assay and a dose-response assay using the IncuCyte^TM^FLR. As shown in Figure 5, bryostatin-1 failed to inhibit ABCB1-mediated efflux of calcein AM in both assays.

**Table 1:** Compounds from the kinase inhibitior library are identified as ABCB1 inhibitors by the high-throughput imaging-based efflux assay.

Name	Target	Name	Target
NVP-TAE684	ALK	NVP-BSK805	JAK
SNS-314 Mesylate	Aurora	Deforolimus (MK-8669)*	mTOR
Nilotinib*	Bcr-Abl	Everolimus (RAD001)*	mTOR
LY2603618	CHK	Rapamycin (Sirolimus)*	mTOR
Imatinib Mesylate*	c-Kit/PDGFR	Temsirolimus*	mTOR
MP-470 (Amuvatinib)	c-Kit/PDGFR/ c-Met	WYE-687	mTOR
Masitinib (AB1010)	c-Kit/PDGFR/ FGFR	WAY-600	mTOR
Ki8751	c-Kit/VEGFR/ PDGFR	Crenolanib (CP-868596)	PDGFR
Pelitinib	EGFR	BEZ235	PI3K/mTOR
WZ3146	EGFR	BI 2536	PLK
WZ4002	EGFR	GSK461364	PLK
Gefitinib (Iressa)*	EGFR/Akt	BI6727	PLK
BIBW2992 (Tovok)	EGFR/HER2	Bosutinib (SKI-606)*	Src
Lapatinib Ditosylate*	EGFR/HER2	AZD0530 (Saracatinib)*	Src/Abl
TG101209	FLT-3/JAK	Cediranib (AZD2171)*	VEGFR
CI-1033 (Canertinib)*	HER2/EGFR	AV-951 (Tivozanib)	VEGFR/ PDGFR
NVP-ADW742	IGF-1R	Imatinib (STI571)*	VEGFR/ PDGFR

* Compounds that have been previously reported to interact with ABCB1. References are listed separately (Text S1).

KB-V1 cells treated with the kinase inhibitor library of 193 compounds at 10 µM and 1 µM calcein AM were examined by the IncuCyte^TM^FLR. The object intensities of each sample were normalized to XR9576 treated cells and expressed as the percentage of inhibition. Compounds that displayed at least 50% inhibition of ABCB1-mediated efflux in all three independent experiments are listed.

**Figure 6:**
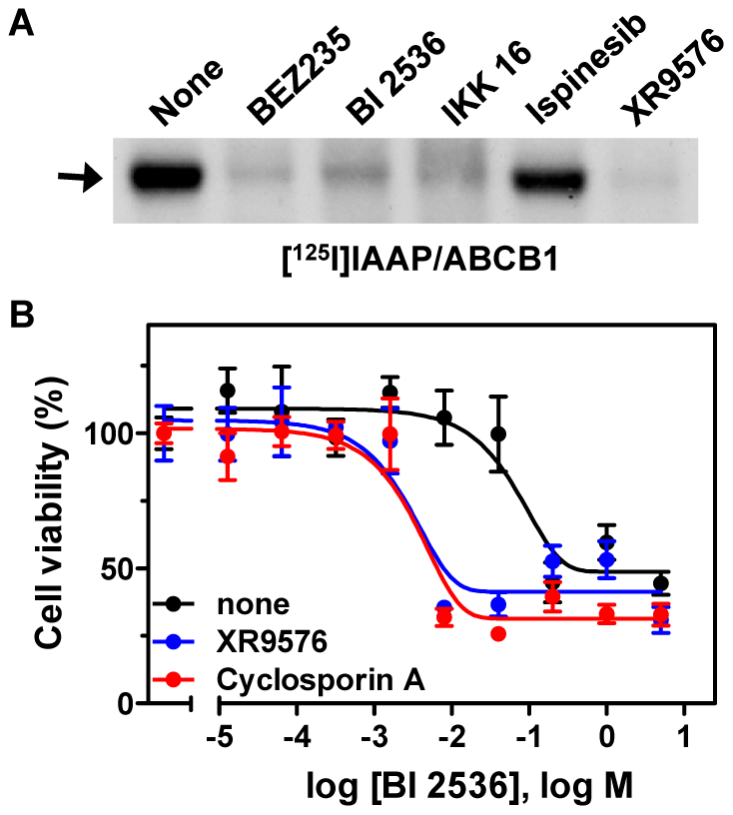
BEZ235, BI 2536, and IKK 16 interfered with binding of ABCB1 and substrate; and inhibition of ABCB1 enhanced drug sensitivities of HCT-15-Pgp cells to BI 2536. **A**. BEZ235, BI 2536, and IKK 16 inhibited [^125^I]IAAP binding to ABCB1. Plasma membranes from Hifive cells overexpressing human ABCB1 were incubated with 20 µM BEZ235, BI 2536, IKK 16, ispinesib, and 5 µM XR9576, then [^125^I]IAAP was added for 10 minutes. The incorporation of [^125^I]IAAP into ABCB1 was shown by autoradiograph. **B**. HCT-15-Pgp cells plated in 96-well plates were treated with BI 2536 in the presence or absence of XR9576 (0.2 µM) and cyclosporin A (5 µM). After 48 hours, the cell viability was evaluated with CCK-8. The relative cell viabilities are presented as mean ±SD (n = 3).

BEZ235, BI 2536, IKK 16, and ispinesib were also tested for their ability to interfere with the direct binding of the radiolabeled-ABCB1 photoaffinity substrate, [^125^I]IAAP, and ABCB1. As shown in Figure 6A, BEZ235, BI 2536, and IKK 16 successfully competed with radiolabeled [^125^I]IAAP for direct binding to ABCB1. However, ispinesib only showed a marginal effect on [^125^I]IAAP-ABCB1 interaction, suggesting a unique mechanism of action. BI 2536, a potent Polo-like kinase inhibitor, was also evaluated in a cytotoxicity assay. As shown in Figure 6B, BI 2536 induced dose-dependent cell death of HCT-15-Pgp cells, an ABCB1-overexpressing cell line. Pre-treatment of HCT-15-Pgp cells with ABCB1 inhibitors, XR9576 and cyclosporin A, before the addition of BI 2536 enhanced the drug sensitivity of the cells to BI 2536, by orders of magnitude as shown in Figure 6B. XR9576 and cyclosporin A reduced the IC_50_ value of BI 2536 from 1.28 µM to 1.4 nM and 0.86 nM, respectively. These results demonstrated that the fluorescent live cell imaging-based high-throughput assay successfully identified a number of new ABCB1 inhibitors using a 384-well plate platform.

## Discussion

ABCB1 is widely recognized for its role in multidrug resistance of cancer cells. In addition to its clinically relevant functions, it also influences the cellular environment and drug-drug interactions in normal cells. In order to advance chemotherapeutic treatment strategies and current pharmacological knowledge of drug-drug interactions, it is essential to discover drugs and new compounds that target ABCB1 transport. Thus, developing new methods and building upon current techniques that can be used for evaluating potential ABCB1 substrates is imperative.

We have developed a high-throughput cell- and imaging-based assay for measuring ABCB1 inhibition via calcein AM efflux using a fluorescent and phase-contrast live cell imaging system, the IncuCyte^TM^FLR. Our method employs the IncuCyte^TM^FLR fluorescent imaging capabilities and software to produce time-sensitive, dose-dependent, reliable, and reproducible results. This modified application of the flow cytometry calcein AM efflux assay [18], [19] can be used to efficiently screen large libraries of natural and synthetic compounds. Though we have employed the technology of the IncuCyte^TM^FLR in our study, this method is platform agnostic and can be performed using any fluorescent microscopic technology with software that can record and quantify fluorescent images.

Unlike flow cytometry-based calcein AM assays, which require cells to be either grown in suspension or detached from culture vessels for treatment with drugs, the fluorescent microscopy-based imaging capacity of the IncuCyte^TM^FLR measures fluorescent calcein in cell monolayers. This enables cells to be plated and treated, then immediately imaged in the same vessels to obtain cellular fluorescence values, which can indicate whether a compound is a potential ABCB1 inhibitor. In addition to the fluorescence values, phase-contrast images allow cell viability and density pre- and post-treatment to be simultaneously compared. This aids in the identification of compounds that are cytotoxic to the cells. Although compounds that auto-fluoresce interfere with fluorescent imaging and cannot be quantitatively analyzed by our assay, this limitation is common in all fluorescent plate reader-based efflux assays. In contrast to the plate reader-based assay, the imaging-based assay provides the opportunity to directly observe the cells for cellular fluorescence. If desired, alternative assays can be performed to further evaluate the compounds.

The live cell imaging-based assay was validated through the examination of known ABCB1 inhibitors, verapamil, cyclosporin A, and XR9576, which all displayed dose-dependent inhibition of ABCB1-mediated efflux. Because our assay does not include wash steps to remove calcein AM from the medium after loading, the accumulation of cellular fluorescent calcein increases with time. The orders in which the wells in the plate are scanned and the position of both positive and negative control wells are critical for the success of this high-throughput assay. The cell imaging-based high-throughput calcein AM efflux assay is dependent on the IncuCyte^TM^FLR recording one image at a time. To scan the tissue culture vessels, the IncuCyte^TM^FLR uses an algorithm that determines the most efficient scanning path. For an entire 384-well plate, the IncuCyte^TM^FLR reads one column at a time starting from one of the four corners, therefore, only an entire 384-well plate should be selected for the high-throughput assays, and both negative and positive controls should be included in each column. Only one plate should be treated and scanned at a time. For a 96-well plate, full or partial, and a partial section of a 384-well plate, the scanning paths do not follow the columns or rows in a set path. Therefore, when performing the efflux assay in 96-well plates, no more than six columns should be scanned to avoid delays in the time-dependent accumulation and measurement of calcein fluorescence in the cells.

In order to validate and assess the robustness of our assay, we selected four compounds that were positive hits in the cell imaging-based assay, BEZ235, BI 2536, IKK 16, and ispinesib, to further confirm their interaction with ABCB1. Each of the four compounds inhibited ABCB1-medicated calcein AM efflux in the flow cytometry assay and displayed dose-dependent inhibition of ABCB1-mediated efflux in our cell imaging-based efflux assay (Figure 5); and all, but ispinesib, also inhibited binding of [^125^I]IAAP, an ABCB1 substrate, to ABCB1, suggesting that BEZ235, BI 2536, and IKK 16 are inhibitors of ABCB1 (Figure 6A). Additional experiments must be performed to elucidate if these compounds are directly transported by ABCB1.

We speculate that ispinesib is an allosteric modulator, or it binds to an alternate drug-binding site on ABCB1, since it inhibited calcein AM efflux but failed to inhibit binding of [^125^I]IAAP to ABCB1. Allosteric modulation of ABCB1 has been described previously [39], [40], [41]. Unlike substrates, which are also used as inhibitors, such as cyclosporin A and verapamil, the allosteric modulator of ABCB1, *cis*-(*Z*)-flupentixol, does not interfere with substrate and [^125^I]IAAP-ABCB1 interaction, instead it changes ABCB1 conformation and prevents substrate translocation and dissociation, resulting in a stable but reversible ABCB1-substrate complex [42]. A novel copper complex, CuNG, was also identified as an ABCB1 modulator that inhibited ABCB1-mediated efflux but did not compete with [^125^I]IAAP for binding to ABCB1 [43]. Further analysis of the interaction between ispinesib and ABCB1 is needed to determine if ispinesib modulates ABCB1 by other mechanisms.

BEZ235, a PI3K/mTOR dual inhibitor, is currently in Phase I and II clinical trials for patients with advanced solid tumors as a single therapeutic agent as well as in combination with other agents [44]. The discovery of BEZ235 as an ABCB1 inhibitor could enhance current knowledge on drug availability of single agents and provide insight into drug-drug interactions that may occur in combination therapies using BEZ235.

BI 2536, a PLK1 inhibitor, has also been tested in clinical trials for treating solid tumors but showed only limited efficacy due to dose-limiting toxicity [45]. It has recently been reported that the reduced efficacy of BI 2536 on the progression of hepatocellular carcinoma is due to low intratumoral drug levels [46]. We found that inhibitors of ABCB1 significantly increased the sensitivity of ABCB1 overexpressing cancer cells to BI 2536 (Figure 6B). Our discovery that BI 2536 is an ABCB1 inhibitor/substrate may shed light on the development of improved therapies that can enhance the efficacy of BI 2536.

Several efflux-based high-throughput assays for screening inhibitors of ABC transporters have been reported in recent years [21], [22], [47], [48], [49], [50]. These assays often use fluorescent substrates as a means of detecting inhibition. Assays that rely on fluorescent plate readers, that are designed to detect homogenous fluorescent signals, are not optimal for detecting fluorescent signals emitted by adherent cells which often display variable cell density in a single well. Although the high-throughput efflux assay based on liquid handling robotics-assisted flow cytometry provides all the benefits associated with the flow cytometry assay, it requires a large number of cells and involves several washing steps, which can be time consuming and could disrupt cells [34]. The cell imaging based high-throughput efflux assay we describe in this report utilizes fluorescent and phase-contrast microscopy-based cell imaging techniques, which exhibit high fluorescent sensitivity and resolution. Our assay is simple and convenient, enabling multiple assays to be performed during a single day. Before image acquisition, our assay only requires two steps: addition of the potential inhibitor immediately followed by addition of the fluorescent substrate (calcein AM).

We demonstrated that the IncuCyte^TM^FLR-based high-throughput calcein AM efflux assay can be used to screen wide ranges of compounds for ABCB1 inhibition and has many advantages over current methods used to identify ABCB1 inhibitors. Identification of compounds that interact with ABCB1 could influence their dose response and therapeutic effectiveness in the setting of appropriate target cells expressing ABCB1. In addition to ABCB1 screening, the techniques of this assay can be readily applied to screen inhibitors for other transporters. The discovery of new ABC transporter inhibitors can lead to advancements in clinical treatments and provide insight into the biological functions of ABC transport proteins.

## Supporting Information

Figure S1 **MitoTracker®Green FM in ABCB1-mediated efflux assay. A**. Flow cytometry-based efflux assay comparing MitoTracker®Green FM (orange and green lines) and calcein AM as fluorescent substrates (red and blue lines). XR9576 was the positive control for ABCB1 inhibition (green and blue lines). **B** and **C**. Cell imaging-based assay of MitoTracker®Green FM efflux using the IncuCyte^TM^FLR imaging system. Mean fluorescence intensities (**B**) and object intensities (**C**) were plotted. Data are mean ± SD (n = 3).
(TIF)

Figure S2 **Comparison of fluorescent plate reader-based and cell imaging-based efflux assays.** KB-V1 cells plated in 96-well plates were treated with increasing concentrations of verapamil (ABCB1 inhibitor) and calcein AM and incubated at 37°C for 1 hour. XR9576 treatment was included as a positive control. The fluorescence intensities of the cells were evaluated by a fluorescent plate reader and the IncuCyte^TM^FLR imaging system. Relative fluorescence intensities were normalized to XR9576 treated cells and plotted.
(TIF)

Figure S3 **The frequency distribution of the Z-factors in the 384-well plate-based efflux assay.** Z-factors from each column of the three 384-well plates were calculated using XR9576/calcein AM treated cells as a positive control and calcein AM only treated cells as a negative control. The frequency distribution histogram was generated with a 0.2 bin using GraphPad Prism.
(TIF)

Text S1 **Supplementary references for** **Table** **1** **.**
(DOCX)
