# Supplementary figures and images for: Screening Compounds with a Novel High-Throughput ABCB1-Mediated Efflux Assay Identifies Drugs with Known Therapeutic Targets at Risk for Multidrug Resistance Interference
Source: PLoS One. 2013 Apr 10;8(4):e60334. doi: 10.1371/journal.pone.0060334 (PMC3622673; doi:10.1371/journal.pone.0060334)

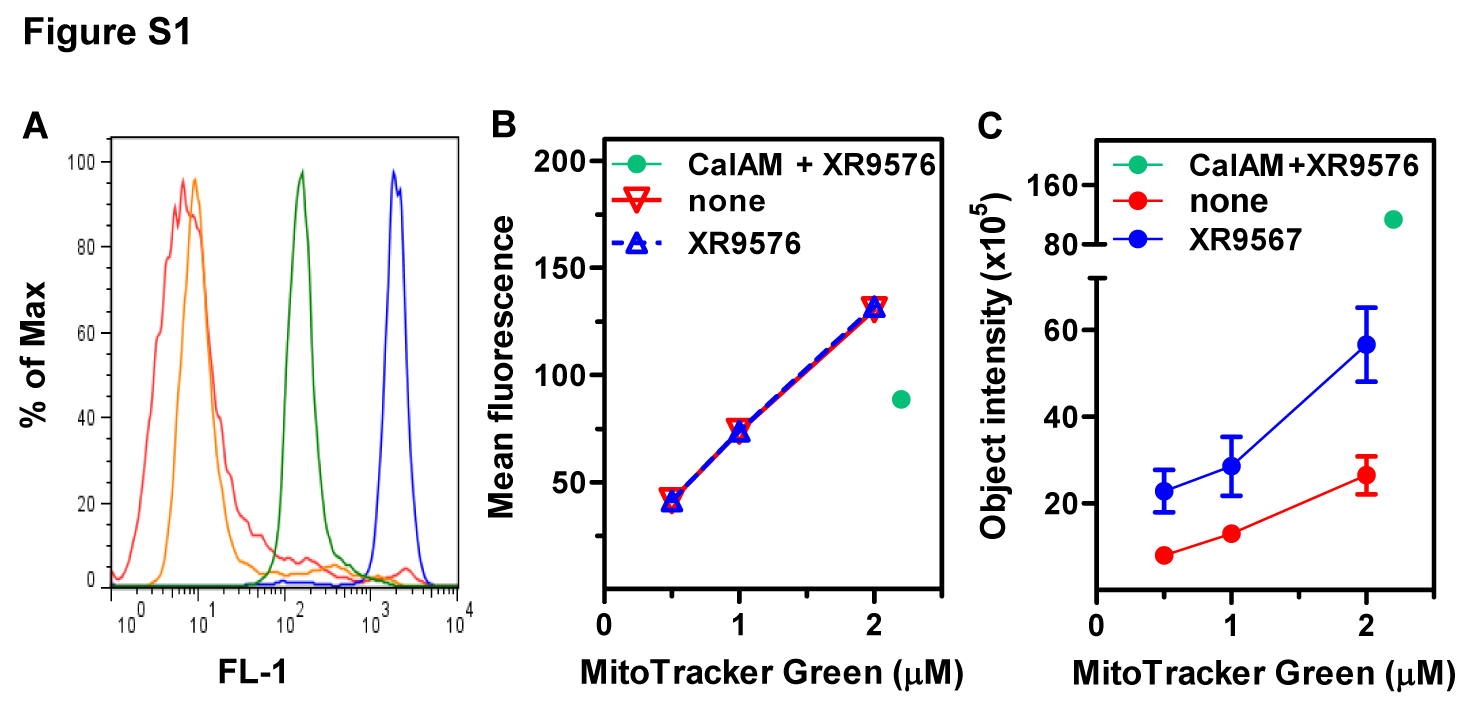

Supplement: Figure S1 — MitoTracker®Green FM in ABCB1-mediated efflux assay. A. Flow cytometry-based efflux assay comparing MitoTracker®Green FM (orange and green lines) and calcein AM as fluorescent substrates (red and blue lines). XR9576 was the positive control for ABCB1 inhibition (green and blue lines). B and C. Cell imaging-based assay of MitoTracker®Green FM efflux using the IncuCyteTMFLR imaging system. Mean fluorescence intensities (B) and object intensities (C) were plotted. Data are mean ± SD (n = 3). (TIF) [file pone.0060334.s001.tif]

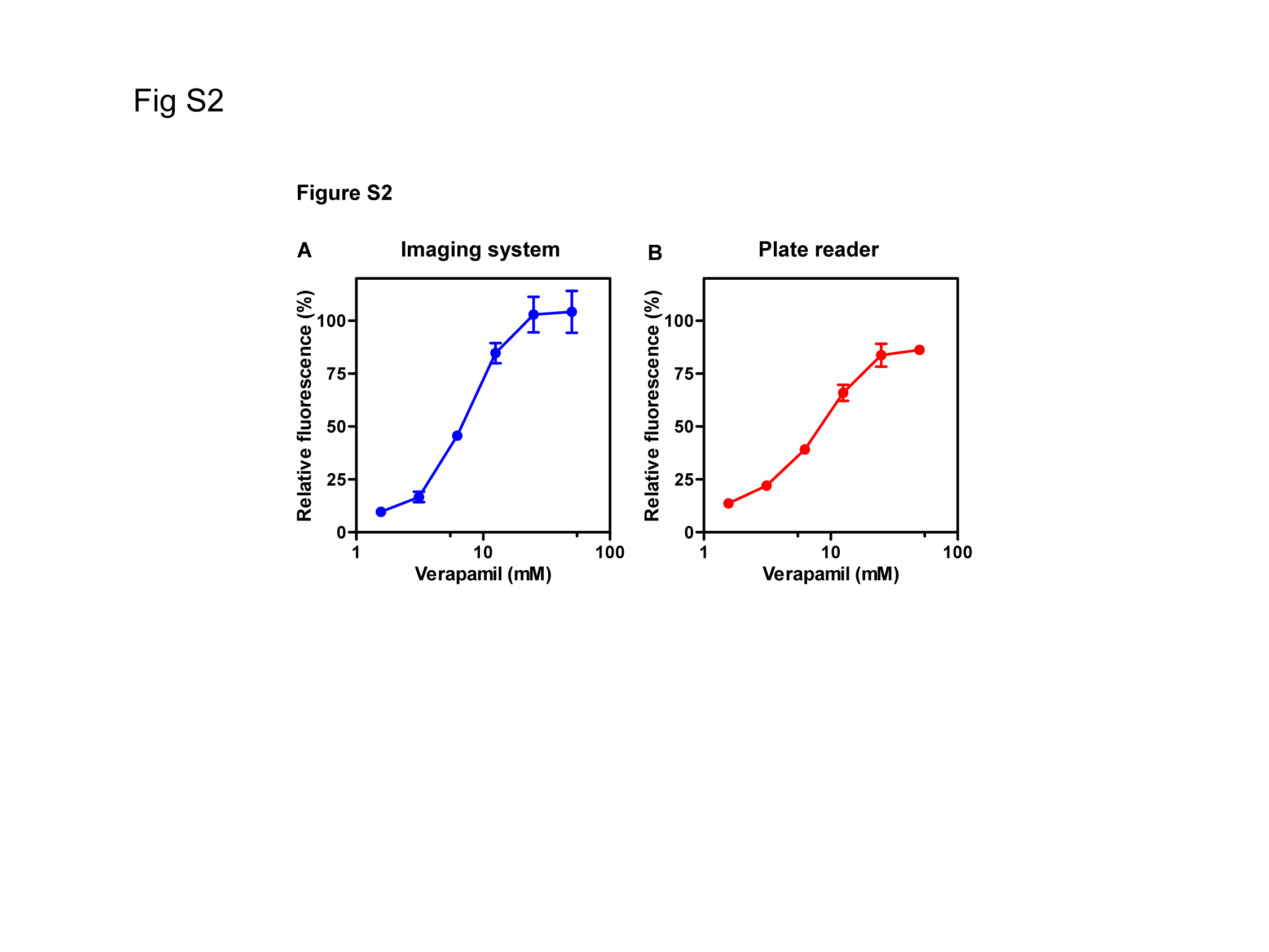

Supplement: Figure S2 — Comparison of fluorescent plate reader-based and cell imaging-based efflux assays. KB-V1 cells plated in 96-well plates were treated with increasing concentrations of verapamil (ABCB1 inhibitor) and calcein AM and incubated at 37°C for 1 hour. XR9576 treatment was included as a positive control. The fluorescence intensities of the cells were evaluated by a fluorescent plate reader and the IncuCyteTMFLR imaging system. Relative fluorescence intensities were normalized to XR9576 treated cells and plotted. (TIF) [file pone.0060334.s002.tif]

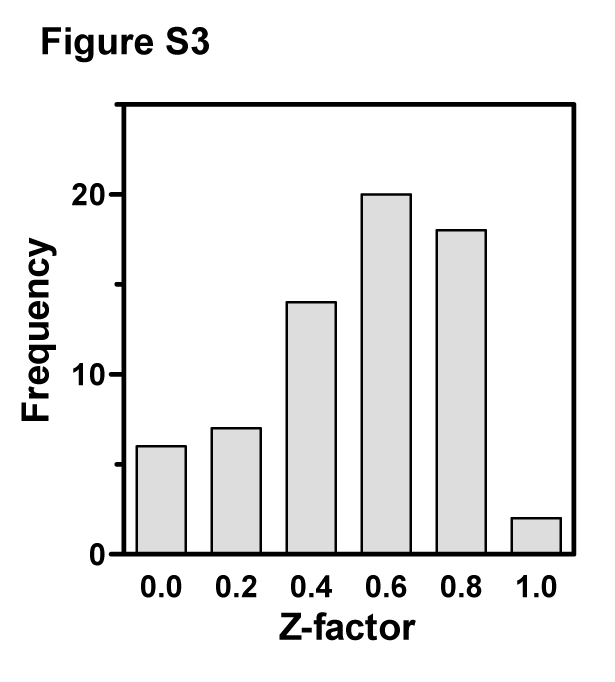

Supplement: Figure S3 — The frequency distribution of the Z-factors in the 384-well plate-based efflux assay. Z-factors from each column of the three 384-well plates were calculated using XR9576/calcein AM treated cells as a positive control and calcein AM only treated cells as a negative control. The frequency distribution histogram was generated with a 0.2 bin using GraphPad Prism. (TIF) [file pone.0060334.s003.tif]
