# Supplementary material for: Screening Compounds with a Novel High-Throughput ABCB1-Mediated Efflux Assay Identifies Drugs with Known Therapeutic Targets at Risk for Multidrug Resistance Interference
Source: PLoS One. 2013 Apr 10;8(4):e60334. doi: 10.1371/journal.pone.0060334 (PMC3622673; doi:10.1371/journal.pone.0060334)
Supplement: Text S1 — Supplementary references for Table 1 . (DOCX) [file pone.0060334.s004.docx]

| **Text S1.**  **Supplementary references for Table 1**  Compounds that have been previously reported to interact with ABCB1:  Gefitinib [1] |
| --- |
| Cl-1033 (Canertinib) [2] |
| AZD0530 (Saracatinib) [3] |
| Nilotinib [4,5] |
| Cediranib (AZD2171)[6] |
| Bosutinib (SKI-606) [7] |
| Imatinib (STI571)[8,9] |
| Imatinib mesylate[10] |
| Deforolimus (Ridaforolimus)[11] |
| Everolimus [12,13] |
| Rapamycin [14,15] |
| Temsirolimus [12] |
| Lapatinib Ditosylate [16] |

1. Kitazaki T, Oka M, Nakamura Y, Tsurutani J, Doi S, et al. (2005) Gefitinib, an EGFR tyrosine kinase inhibitor, directly inhibits the function of P-glycoprotein in multidrug resistant cancer cells. Lung Cancer 49: 337-343.

2. Minocha M, Khurana V, Qin B, Pal D, Mitra AK (2012) Enhanced brain accumulation of pazopanib by modulating P-gp and Bcrp1 mediated efflux with canertinib or erlotinib. Int J Pharm 436: 127-134.

3. Liu KJ, He JH, Su XD, Sim HM, Xie JD, et al. (2013) Saracatinib (AZD0530) is a potent modulator of ABCB1-mediated multidrug resistance in vitro and in vivo. Int J Cancer 132: 224-235.

4. Tiwari AK, Sodani K, Wang SR, Kuang YH, Ashby CR, Jr., et al. (2009) Nilotinib (AMN107, Tasigna) reverses multidrug resistance by inhibiting the activity of the ABCB1/Pgp and ABCG2/BCRP/MXR transporters. Biochem Pharmacol 78: 153-161.

5. Shukla S, Skoumbourdis AP, Walsh MJ, Hartz AM, Fung KL, et al. (2011) Synthesis and characterization of a BODIPY conjugate of the BCR-ABL kinase inhibitor Tasigna (nilotinib): evidence for transport of Tasigna and its fluorescent derivative by ABC drug transporters. Mol Pharm 8: 1292-1302.

6. Tao LY, Liang YJ, Wang F, Chen LM, Yan YY, et al. (2009) Cediranib (recentin, AZD2171) reverses ABCB1- and ABCC1-mediated multidrug resistance by inhibition of their transport function. Cancer Chemother Pharmacol 64: 961-969.

7. Hegedus C, Ozvegy-Laczka C, Apati A, Magocsi M, Nemet K, et al. (2009) Interaction of nilotinib, dasatinib and bosutinib with ABCB1 and ABCG2: implications for altered anti-cancer effects and pharmacological properties. Br J Pharmacol 158: 1153-1164.

8. Czyzewski K, Styczynski J (2009) Imatinib is a substrate for various multidrug resistance proteins. Neoplasma 56: 202-207.

9. Dai H, Marbach P, Lemaire M, Hayes M, Elmquist WF (2003) Distribution of STI-571 to the brain is limited by P-glycoprotein-mediated efflux. J Pharmacol Exp Ther 304: 1085-1092.

10. Breedveld P, Pluim D, Cipriani G, Wielinga P, van Tellingen O, et al. (2005) The effect of Bcrp1 (Abcg2) on the in vivo pharmacokinetics and brain penetration of imatinib mesylate (Gleevec): implications for the use of breast cancer resistance protein and P-glycoprotein inhibitors to enable the brain penetration of imatinib in patients. Cancer Res 65: 2577-2582.

11. Stroh M, Palcza J, McCrea J, Marsilio S, Breidinger S, et al. (2012) The effect of multiple doses of rifampin and ketoconazole on the single-dose pharmacokinetics of ridaforolimus. Cancer Chemother Pharmacol 69: 1247-1253.

12. Minocha M, Khurana V, Qin B, Pal D, Mitra AK (2012) Co-administration strategy to enhance brain accumulation of vandetanib by modulating P-glycoprotein (P-gp/Abcb1) and breast cancer resistance protein (Bcrp1/Abcg2) mediated efflux with m-TOR inhibitors. Int J Pharm 434: 306-314.

13. Galanis E, Buckner JC, Maurer MJ, Kreisberg JI, Ballman K, et al. (2005) Phase II trial of temsirolimus (CCI-779) in recurrent glioblastoma multiforme: a North Central Cancer Treatment Group Study. J Clin Oncol 23: 5294-5304.

14. Arceci RJ, Stieglitz K, Bierer BE (1992) Immunosuppressants FK506 and rapamycin function as reversal agents of the multidrug resistance phenotype. Blood 80: 1528-1536.

15. Pawarode A, Shukla S, Minderman H, Fricke SM, Pinder EM, et al. (2007) Differential effects of the immunosuppressive agents cyclosporin A, tacrolimus and sirolimus on drug transport by multidrug resistance proteins. Cancer Chemother Pharmacol 60: 179-188.

16. Molina JR, Kaufmann SH, Reid JM, Rubin SD, Galvez-Peralta M, et al. (2008) Evaluation of lapatinib and topotecan combination therapy: tissue culture, murine xenograft, and phase I clinical trial data. Clin Cancer Res 14: 7900-7908.
